# From bulk samples to single cells: measurement strategies in Raman-based antibiotic susceptibility testing

**DOI:** 10.1007/s00216-026-06558-3

**Published:** 2026-06-03

**Authors:** Marie-Luise Enghardt, Andreea Winterfeld, Susanne Pahlow, Anja Silge, Jürgen Popp

**Affiliations:** 1 Member of: Leibniz Research Alliance Health Technologies; Leibniz Research Alliance INFECTIONS; Leibniz Centre for Photonics in Infection Research (LPI), Leibniz Institute of Photonic Technology, Jena, Germany; 2 Institute of Physical Chemistry (IPC) and Abbe Center of Photonics (ACP), Member of the Leibniz Centre for Photonics in Infection Research (LPI), Friedrich Schiller University Jena, Helmholtzweg 4, 07743 Jena, Germany; 3 InfectoGnostics Research Campus Jena, Center of Applied Research, Philosophenweg 7, 07743 Jena, Germany

**Keywords:** Raman spectroscopy, Antibiotic susceptibility testing, Single-cell Raman spectroscopy, *Escherichia coli*, Bioassays

## Abstract

Raman-spectroscopy based antibiotic susceptibility testing (AST) enables rapid phenotypic analysis of bacterial responses to antibiotics. However, many established approaches rely on bulk bacterial material, limiting their applicability to samples with low and variable cell numbers, such as urine, where washing or medium exchange is often impractical. We systematically compare three Raman-based measurement strategies differing in sample preparation and cellular presentation: (i) dried bulk measurements on CaF_2_ substrates after centrifugation, (ii) cell cloud measurements in liquid using a dielectrophoresis (DEP) chip, and (iii) single-cell measurements on metal-coated filters after filtration. *Escherichia coli* ATCC 25922 was exposed to amoxicillin and levofloxacin across defined concentration ranges. Raman spectra were analysed using a PCA-LDA to classify between bacterial growth and inhibition, with validation against broth microdilution (BMD). Dried bulk measurements showed the highest agreement with BMD and the most robust classification performance, but require centrifugation and washing, limiting their suitability for low bacterial cell numbers. DEP-chip measurements enabled analysis in a closed system with reduced handling while maintaining good predictive performance, although they depend on sufficient cell density and involve higher chip costs. Single-cell measurements provided the simplest workflow and allowed analysis at clinically relevant low bacteria concentrations but showed reduced classification robustness due to increased spectral heterogeneity and longer acquisition times. Overall, this study highlights methodological trade-offs between robustness, experimental complexity, and suitability for low-cell-number samples. These findings provide practical guidance for selecting appropriate Raman-based AST strategies depending on sample type and application.

**Graphical abstract:**

**Figure:**
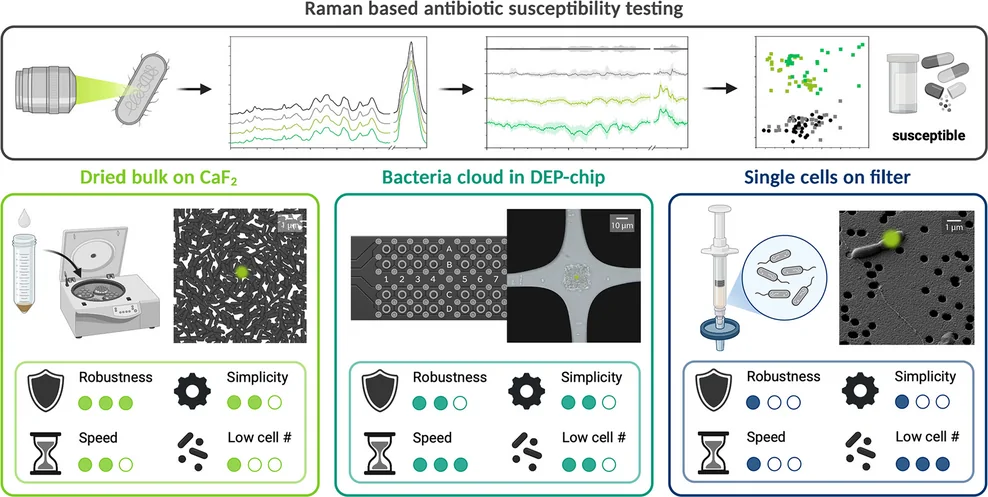

**Supplementary Information:**

The online version contains supplementary material available at https://doi.org/10.1007/s00216-026-06558-3.

## Introduction

Antimicrobial resistance (AMR) represents a significant global health threat and was associated with an estimated 4.95 million deaths worldwide in 2019 [1]. Rapid determination of bacterial susceptibility to antibiotics is therefore essential to initiate effective treatment. Conventional phenotypic methods for antibiotic susceptibility testing (AST), such as broth microdilution, are based on bacterial growth and typically take 18–24 h to produce results [2]. This delay often leads to empirical antibiotic therapy and can contribute to inappropriate antibiotic use [3]. Molecular resistance tests can provide faster results, for example by detecting known resistance determinants using PCR-based assays. However, such approaches are limited to previously characterized mechanisms and do not directly reflect the phenotypic antibiotic response of bacterial cells [3, 4]. Several alternative approaches for rapid antibiotic susceptibility testing have been proposed, but many of these methods rely on complex equipment, require specialized workflows or labels for sample preparation, and are difficult to implement in routine clinical settings [3]. In addition to analytical performance, practical constraints such as cost, ease of handling, or compatibility with decentralized clinical settings are relevant for real-world implementation.

Raman spectroscopy has established itself as a promising optical approach for the rapid phenotypic characterization of bacterial cells. Raman spectra provide molecular fingerprints that reflect the biochemical composition of bacterial cells and enable label-free characterization [5–7]. Raman-based AST approaches typically rely on monitoring antibiotic-induced phenotypic changes by comparing treated samples to untreated controls, often at concentrations around the expected minimum inhibitory concentration (MIC), thereby enabling classification of bacterial growth versus inhibition within a few hours.

Therefore, various experimental strategies have therefore been developed, which can be divided into measurements on cell ensembles and single-cell Raman spectroscopy. Bulk approaches, including measurements on concentrated or dried bacterial samples, yield stable spectra with a high signal-to-noise ratio and enable robust chemometric analysis [8]. In contrast, Raman measurements in liquid environments can be performed using microfluidic or dielectrophoresis (DEP)-based platforms to trap or concentrate bacterial cells prior to spectral acquisition [9]. Furthermore, single-cell Raman spectroscopy enables the investigation of individual bacterial cells and the analysis of phenotypic heterogeneity within bacterial populations [10]. Importantly, the chosen sample preparation strategy not only affects handling and cell recovery, but also determines the physical presentation of bacterial cells during Raman measurement, such as bulk deposits, cell clusters, or individual cells, which in turn influences spectral characteristics and classification performance. In addition, aspects such as system sterility, compatibility with portable instrumentation, and integration into closed workflows represent important considerations for clinical translation.

However, before Raman-based antibiotic susceptibility testing can be adopted into routine clinical practice, the key question of how bacterial samples should be prepared for measurement must be resolved. Sample preparation represents a critical bottleneck, as it has a direct impact on spectral quality, reproducibility, the risk of contamination, suitability for low cell counts, and the complexity of the entire workflow. Ideally, a preparation strategy should therefore be quick, simple, robust, and cost-effective, while at the same time providing reliable phenotypic information. It remains unclear which measurement strategy via Raman spectroscopy offers the best balance between analytical performance and practical applicability.

In this study, three representative Raman-based measurement strategies resulting from different sample preparation strategies were directly compared: measurements on dried bacterial samples, measurements in liquid using an advanced DEP-chip platform (see [9]), and single-cell measurements on an aluminium-coated filter substrate. *Escherichia coli* was investigated as a model organism following exposure to two clinically relevant antibiotics with different modes of action, amoxicillin and levofloxacin, in order to capture different phenotypic response patterns. By systematically evaluating these approaches under comparable conditions, this work aims to identify key methodological trade-offs and assess which combination of sample preparation and measurement strategy represents the most suitable compromise for a rapid, clinically relevant Raman-based antibiotic susceptibility test.

## Materials and methods

### Bacterial strain

The Gram-negative *E. coli* ATCC 25922 was propagated in brain-heart infusion medium (BHI, brain heart infusion CM135, Oxoid) until the exponential phase, harvested, and then stored at −80 °C in BHI supplemented with 20% glycerol until use.

### Pre-cultivation and antibiotic treatment

For each experiment, 300 µl of cryogenic stock solution was inoculated into 50% diluted antibiotic test medium 2 (AT2 50%) and incubated for 2–3 h in baffled flasks (Carl Roth LY94.1) prior to antibiotic treatment. AT2 50% was used as a Raman-compatible liquid medium due to its low intrinsic fluorescence background. The medium consisted of 5 g/l NaCl (≥ 99%, Carl Roth 3957.1), 3 g/l tryptone peptone (Carl Roth 6681.1), and 0.1 g/l MgSO_4_ × 7 H_2_O (Sigma 63138), which were dissolved in deionized water and sterilized by autoclaving. After cooling, glucose (Sigma G7528) was added to reach 5% v/v and the medium was diluted to 50% with one quarter DPBS (1 ×, Carl Roth 9124.1) and one quarter sterile, deionized water. After pre-cultivation, the bacterial suspensions were transferred to baffled flasks and adjusted to an OD of 0.08. For this purpose, AT2 50% medium was added to the culture with the appropriate amount of antibiotic to achieve the desired concentration, and the final volume was adjusted to 10 ml per flask. The antibiotic concentrations used were selected based on the minimum inhibitory concentration (MIC), which was determined independently by broth microdilution (BMD) under identical medium conditions. The interaction time between bacteria and antibiotics was set at 90 min for all experiments before Raman measurements were performed.

### Antibiotics

Levofloxacin hemihydrate (CAS No. 138199-71-0, USP, Rockville, MD, USA) and Amoxicillin trihydrate (CAS No. 26787-78-0, USP, Rockville, MD, USA) were weighed and dissolved in sterile, deionized water to prepare 1 g/l stock solutions. The solutions were aliquoted and stored at −20 °C until use.

### MIC estimation for the two investigated antibiotics

Since AT2 50% was used as the growth medium for *E. coli* ATCC 25922 in all experiments, the minimum inhibitory concentrations (MIC) of the antibiotics tested were determined under identical medium conditions. The bacterial suspension used for antibiotic exposure was adjusted to an optical density of OD600 = 0.08. Based on an experimentally determined OD-CFU correlation, this optical density corresponded to approximately 3 × 10^7^ CFU/ml (see SI1). Broth microdilution (BMD) tests were performed for *E. coli* ATCC 25922 with levofloxacin and amoxicillin in concentration ranges from 0.004 to 32 mg/l (see SI2). For the Raman-based AST experiments, the antibiotic concentrations were selected relative to the MIC determined for the strain in AT2 50% media. Four concentrations were used for each AST experiment: two below the MIC (untreated control and MIC – 1 = sub-inhibitory) and two above the MIC (MIC + 1 = inhibitory and MIC + 5 = inhibition control).

### Sample preparation for Raman determination

#### Dry bulk measurements

After 90 min of bacteria-antibiotic interaction, the cells were harvested by centrifugation at 4.000 rpm (1.735 rcf) for 5 min using the Hettich Rotina 380R centrifuge (r = 9.7 cm). The supernatant was discarded, and the pellet was washed twice by centrifugation at 13.300 rpm (11.900 rcf) for 1.5 min using the Eppendorf mini-Spin centrifuge (r = 6 cm), resuspending the cells each time in 1.0 ml of sterile dH_2_O. After the final wash step, the cells were resuspended in the remaining minimal volume. Prior to sample application, the CaF_2_ slides were rinsed with ethanol and sterile water and dried. Subsequently, 3 µl of the concentrated bacterial suspension was applied to the substrate and completely air-dried prior to Raman measurements.

#### DEP chip measurements

The RamanBioAssay (RBA) chip used in this study was manufactured in-house in the clean room facility of the Leibniz Institute of Photonic Technology (Jena, Germany). The microfluidic chip contains a multiwell layout with 40 cavities in which bacteria are enriched by negative dielectrophoresis (DEP), enabling the formation of localized bacterial clusters (“bacterial clouds”) consisting of several hundred cells. The functionality of DEP-based enrichment concepts for Raman measurements has already been applied for a similar chip version [9]. For each measurement, 4 µl of bacterial suspension (3 × 10^7^ CFU/ml) was added to individual wells. The multi-well configuration allows different antibiotic concentrations or samples to be loaded independently onto the same chip. After loading, the chip was sealed with a microplate sealing film (ThermalSeal RT™, Excel Scientific Inc., USA), mounted on the microscope stage and connected to the electrical interface. An alternating electric field (6 V peak-to-peak, 1 MHz) was applied to induce DEP-based trapping of bacterial cells within the quadrupole electrode structures. The enrichment process was monitored microscopically, and within approximately 5 to 15 min, the accumulated bacterial clouds reached diameters of ≥ 10 µm, which were then used as Raman measurement positions.

#### Single-cell measurements

**Fig. 1:**
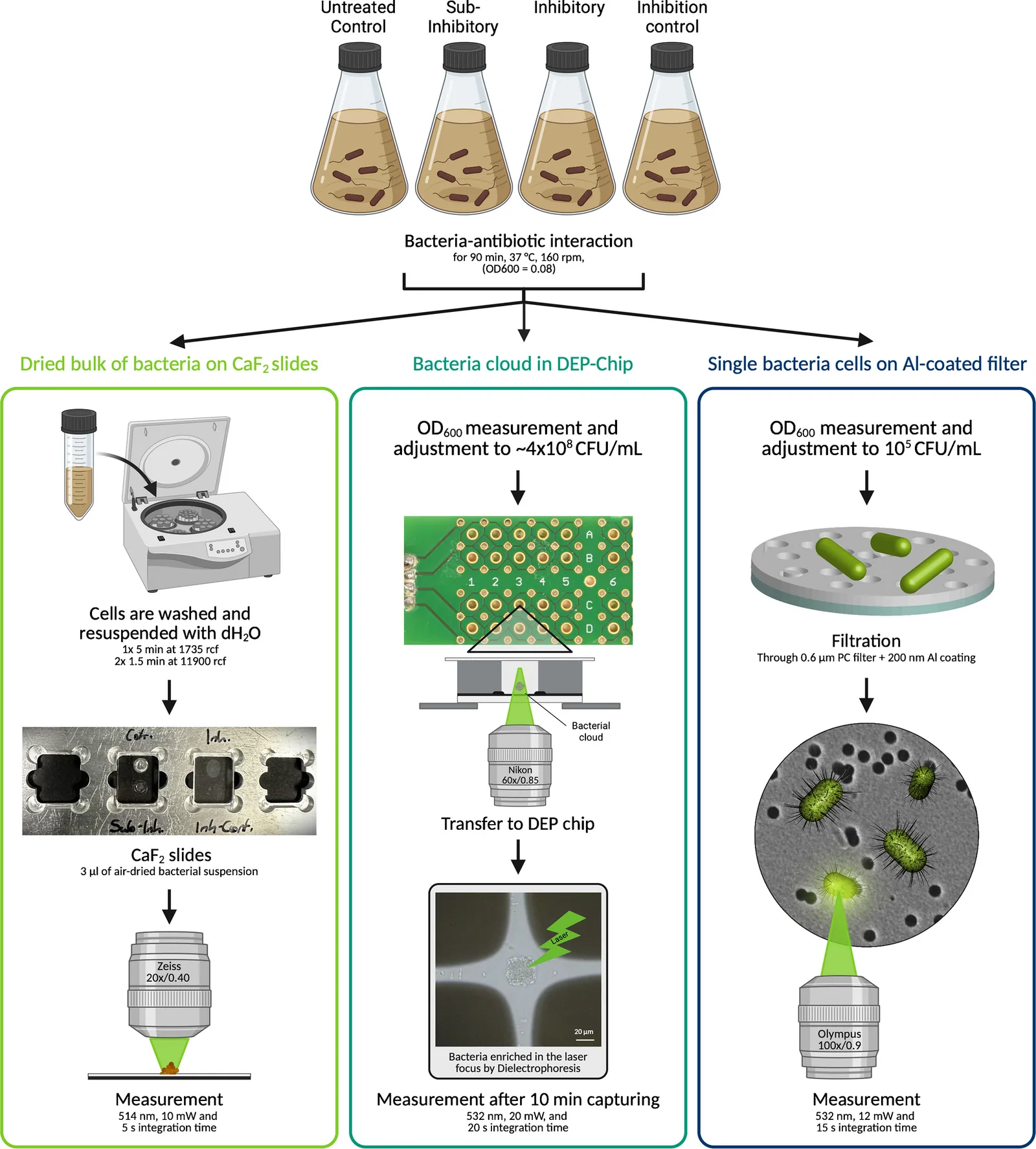
Overview of the three Raman measurement strategies investigated for rapid antibiotic susceptibility testing. The bacteria were exposed to antibiotics at four different concentrations during a 90-min bacteria-antibiotic interaction. Following incubation, the samples were analysed using three different Raman measurement methods. Left: Raman measurements on dried bacterial cells. The cells were harvested by centrifugation, washed twice, concentrated on CaF_2_ slides and air-dried. Centre: Measurements in liquid using a DEP-chip. Bacterial suspensions were adjusted to approximately 3 × 10^7^ CFU/ml, transferred to the DEP chip and concentrated into bacterial clouds by negative dielectrophoresis. Right: Single-cell measurements on Al-coated filters. The samples were adjusted to approximately 10^5^ CFU/ml and filtered through an Al-coated polycarbonate membrane (pore size 0.6 µm), which enabled the measurement of individual bacterial cells. Created with Biorender.com

After the washing steps, however, the cells were resuspended in sterile dH_2_O and a new OD600 measurement was performed to account for any potential cell count change as a result of the washing. Upon this, the solutions were diluted to obtain 1 ml of a concentration of 10^5^ CFU/ml for each investigated mixture. The resulting solutions were then filtered through an in-house fabricated filter membrane (Merck Millipore, polycarbonate membrane filter, 0.6 μm pore size, DTTP01300 coated with 200 nm Al) and let to air dry under ambient conditions. An overview of the three Raman measurement strategies investigated in this study is shown in Figure 1.

### Validation via broth microdilution test (BMD)

A BMD was performed as an internal reference method for validating the RBA-based AST results. For this purpose, bacterial-antibiotic suspensions from the interaction flasks were transferred to a 96-well microtiter plate (Greiner Bio-One, 655161). Four technical replicates of 200 µl were pipetted for each antibiotic concentration. A sterile control with AT2 50% medium was included on each plate. The plates were incubated at 37 °C. During incubation, the optical density at 600 nm was recorded using a FLUOstar Omega microplate reader (BMG LABTECH, Ortenberg, Germany). Every 5 min, the plates were shaken for 30 s with double orbital motion at 700 rpm, followed by a settling time of 0.5 s. Signal acquisition was performed at 22 flashes per well and measurement cycle. Bacterial growth was assessed after 20 h based on visible turbidity compared to the growth control according to [11] criteria for growth interpretation. BMD measurements were performed in parallel for each biological replicate used in the Raman experiments (see SI3). During the validation of the levofloxacin experiments, two biological batches showed no detectable growth at the originally assumed subinhibitory concentration (MIC - 1). No corresponding parallel BMD data was available for the third batch. Therefore, three additional independent biological replicates were analysed to verify this observation. These measurements confirmed the absence of growth at this concentration. Consequently, the classification of this concentration was corrected accordingly for the subsequent data analysis.

### Raman spectroscopic investigation

#### Dry bulk measurements

The dried samples were analysed using a WITec alpha300R Raman microspectrometer (WITec GmbH, Ulm, Germany). Excitation was provided by a diode-pumped solid-state laser (Cobolt, Solna, Sweden) with a wavelength of 514 nm and a nominal maximum output power of 100 mW. The Raman spectra were recorded with a laser power of 10 mW in front of the objective and an integration time of 5 s per spectrum. The Raman signal was collected with a Zeiss EC Epiplan DIC objective (20x magnification, NA=0.4, working distance 3.2 mm) to focus on the dried bacterial layer. For each strain-antibiotic combination, 10 spectra were recorded from untreated and antibiotic-exposed samples at different positions across the deposited bacterial material. Spectral dispersion was achieved using a diffraction grating with 600 lines/mm, resulting in a spectral resolution of approximately 5 cm⁻^1^. The Raman signal was detected at −60 °C using a thermoelectrically cooled, back-illuminated deep depletion CCD detector (DV401A-BV-352, Andor) with 1024×127 pixels and a pixel size of 26 µm×26 µm. Based on the estimation introduced in SI5, the effective confocal probe volume at an excitation of 514 nm corresponded to approximately 2.16 µm in the lateral direction and 14.26 µm in the axial direction.

#### DEP chip measurements

Raman measurements in the DEP Chip were performed using an inverted WITec alpha300 Ri Raman microspectrometer (WITec GmbH, Ulm, Germany). A diode-pumped solid-state laser with an excitation wavelength of 532 nm (DPSS, single longitudinal mode, nominal output power 75 mW) was used as excitation source. The laser power at the sample was adjusted using a software-controlled absolute laser power regulation (TruePower; 0.1 mW setting accuracy) and was set to 20 mW before the objective. The excitation beam was fibre-coupled via a 3 m polarization-maintaining single-mode fibre with FC/APC connection. Raman spectra were acquired in backscattering geometry through the sealed DEP-RBA chip using a Nikon CFI Plan Fluor objective (60× magnification, NA=0.85, working distance 0.31 mm) equipped with a variable correction collar (0.11–0.23 mm). The correction collar was set to 0.20 mm, matching the thickness of the quartz glass of the DEP chip. Based on the estimation introduced in SI5, the effective confocal scanning dimensions were estimated to approximately 1.08 µm laterally and 3.42 µm axially for excitation at 532 nm. Spectra were recorded with an integration time of 20 s. At each measurement position, three consecutive spectra were acquired and averaged. Spectral dispersion was achieved using a 300 lines/mm diffraction grating, providing a spectral resolution of approximately 10 cm⁻^1^ at 532 nm. The Raman signal was coupled into the spectrometer via FC/APC fibre coupling and detected with a thermoelectrically cooled, back-illuminated deep-depletion CCD detector (1024×127 pixels, pixel size 26 µm×26 µm), operated at −60 °C.

#### Single-cell measurements

**Fig. 2:**
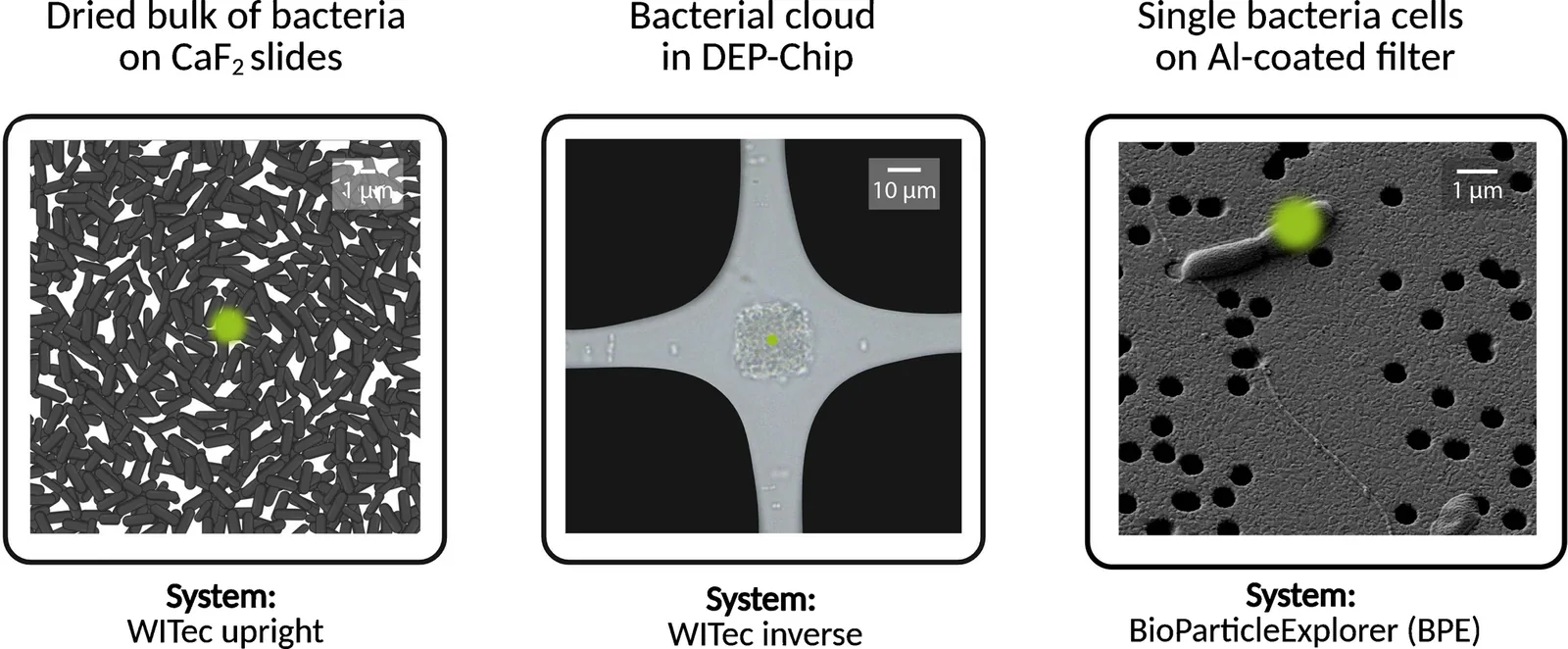
Schematic representation of the cell presentation in the Raman measurements for the three strategies used in this study and the visualization of the effective confocal scanning volumes of different Raman systems. Left: Bulk Raman measurements on dried bacteria on CaF_2_ slides, recorded using an upright WITec Raman microscope. Due to the large axial extent of the confocal volume (Δx,y ~ 2.16 µm, Δz ~ 14.26 µm) and the dense multilayer of dried cells, several bacterial cells contribute simultaneously to the recorded spectrum. Centre: Raman measurements of bacteria concentrated in a bacterial cloud within a DEP chip, using an inverse WITec Raman microscope. Although the confocal volume is smaller (Δx,y ~ 1.08 µm, Δz ~ 3.42 µm), several bacterial cells are located within the dielectrophoretically trapped cluster and can traverse the excitation volume during acquisition, resulting in spectra that represent a heterogeneous image of cells in liquid. Right: Single-cell Raman measurements of bacteria immobilized on Al-coated filters, using the BioParticleExplorer system. The confocal volume (Δx,y ~ 1.02 µm, Δz ~ 3.05 µm) is comparable to the size of a single bacterium, enabling targeted measurements of individual cells.

The single-cell Raman measurements were performed under ambient conditions with a commercially available Raman microscope setup (Bio Particle Explorer, rap.ID Particle Systems GmbH, Berlin, Germany) [12] using a 532 nm solid-state frequency-doubled Nd:YAG laser (LCM-s-111-NNP25, Laser-export Company Ltd.). The laser beam was focused through an Olympus MPLFLN-BD objective (100x magnification, NA=0.9, working distance 1 mm) and a lateral resolution below 1 μm. The incident laser power on the sample was approximately 12 mW. The integration time per spectrum was 15 s. A single-stage monochromator (HE532, Horiba Jobin Yvon, grating of 920 lines) dispersed the backscattered Raman light before its detection with a thermoelectrically cooled CCD (DV 401_BV, Andor Technology) resulting in a spectral resolution of approximately 8 cm^−1^. Based on the estimation introduced in SI5, the effective confocal scanning dimensions are estimated at approximately 1.02 nm in the lateral direction and 3.05 nm in the axial direction for excitation at 532 nm. A minimum of 40 spectra were recorded for each measured substrate. A schematic comparison of the three Raman measurement configurations and their effective confocal sampling volumes is shown in Figure 2.

### Data analysis

Spectral preprocessing and analysis were performed using Ramanmetrix software Version 0.6.13 [13]. The processing workflow included the removal of cosmic spikes using the one-dimensional Laplacian method, wave number calibration based on 4-acetamidophen, baseline correction using the Sensitive Nonlinear Iterative Peak Clipping (SNIP) algorithm, and vector normalization [13].

The exact preprocessing parameters were adjusted in Ramanmetrix on a device-specific manner to account for the different spectral characteristics of the measurement systems. For the data analysis of the single-cell measurements alone, quality filters were also applied to automatically and user-independent filter out the low-quality spectra. For this, the Ramanmetrix software analysed the vibrations of C-H bonds in the 2800–3050 cm^−1^ spectral range and filtered out the spectra that did not meet a specific threshold. In the amoxicillin dataset, the min intensity threshold was set at 5.645. In the case of the levofloxacin dataset, the min intensity threshold was set at 44.45 and the correlation after preprocessing threshold was set at 0.9508. Upon this, all the different resulting datasets were analysed according to the same unified procedure. To isolate the spectral changes associated with antibiotic exposure, difference spectra were calculated. For this purpose, the mean Raman spectrum of the untreated control was subtracted from the spectra obtained under antibiotic treatment. This subtraction was performed separately for each batch, antibiotic, and measurement strategy. The resulting difference spectra highlight spectral changes associated with the bacterial response to antibiotic exposure. At concentrations where bacterial growth occurred, the spectral differences compared to the untreated control remained small, whereas under conditions where bacterial growth was inhibited, stronger spectral deviations were observed. To quantitatively evaluate these effects, the difference spectra were subjected to principal component analysis (PCA) to capture the main causes of variance associated with antibiotic resistance. The resulting principal component scores were then used to train a linear discriminant analysis (LDA) classifier. This model projected the data onto a single discriminant axis and enabled classification into the two phenotypic categories “growth” and “no growth”.

## Results and discussion

It is generally accepted that the successful application of Raman spectroscopy for identification of bacterial species, as well as for phenotypic antibiotic susceptibility testing (AST), necessitates the incorporation of appropriate sample preparation techniques. Depending on the specific choice, the resulting sample with the bacterial cells can have significantly different properties, which also impacts the Raman measurements. Within our study we combined centrifugation, dielectrophoresis and filtration with a Raman spectroscopic antibiotic susceptibility assay. Centrifugation yielded highly dense cell pellets, which we investigated in a dried state. A dielectrophoresis chip generated looser clouds of cells allowing Raman measurements in liquid medium. Filtration with a Raman-compatible metal-coated filter substrate resulted in single cells available for a Raman-spectroscopic characterization.

To enable a consistent and scientifically meaningful comparison, all experiments were conducted using a unified protocol for bacterial cultivation and antibiotic exposure, while only the final sample preparation step was adapted to the respective measurement approach. *Escherichia coli* ATCC 25922 was exposed to amoxicillin and levofloxacin at four concentrations, including sub-inhibitory and inhibitory conditions, as well as an untreated control. Raman-based classification of bacterial growth versus inhibition was performed using supervised multivariate analysis. To validate the Raman-derived results, conventional growth assessment was conducted in parallel using OD600 measurements after extended incubation, serving as the reference standard. It should be noted that different Raman substrates were employed for each measurement mode. The substrate material is closely linked to the distinct physical and optical constraints of each approach, which makes the use of an identical substrate across all methods impractical. For dried bulk measurements, CaF_2_ was selected as it is widely used in Raman studies of biological samples and provides a very low spectral background. However, CaF_2_ is relatively expensive. More cost-efficient alternatives such as fused silica/quartz, aluminium-based substrates, or silicon wafers may be suitable depending on the required spectral range and sensitivity, although potential trade-offs in background contributions and spectral interference need to be considered. In addition, CaF_2_ substrates can be reused after thorough cleaning and sterilization, resulting in a more favourable cost-per-use ratio.

Model performance was evaluated using cross-validation across independent biological replicates. Classification was based on spectral differences between treated and untreated samples, enabling the identification of antibiotic-induced phenotypic changes. The resulting predictive performance and methodological trade-offs of the different measurement strategies are discussed in the following sections.

### Amoxicillin

Amoxicillin, a β-lactam antibiotic, inhibits bacterial cell wall synthesis by binding to penicillin-binding proteins, thereby preventing proper cross-linking of the peptidoglycan layer [14]. This disruption leads to structural instability, activation of autolytic processes, and ultimately cell lysis. Based on this mode of action, we expect pronounced phenotypic changes between growing and inhibited cells, which should be detectable by Raman spectroscopy as differences in their biochemical composition.

A broth microdilution series was prepared to obtain the ground truth for classifying the samples with different antibiotic concentrations into “growth” vs. “no growth” (Figure SI 3.1a). Untreated and sub-inhibitory samples were confirmed as “grown”, whereas inhibitory and inhibition-control samples showed no growth across all biological replicates. These reference classifications were used to train and validate the Raman-based models.

**Fig. 3:**
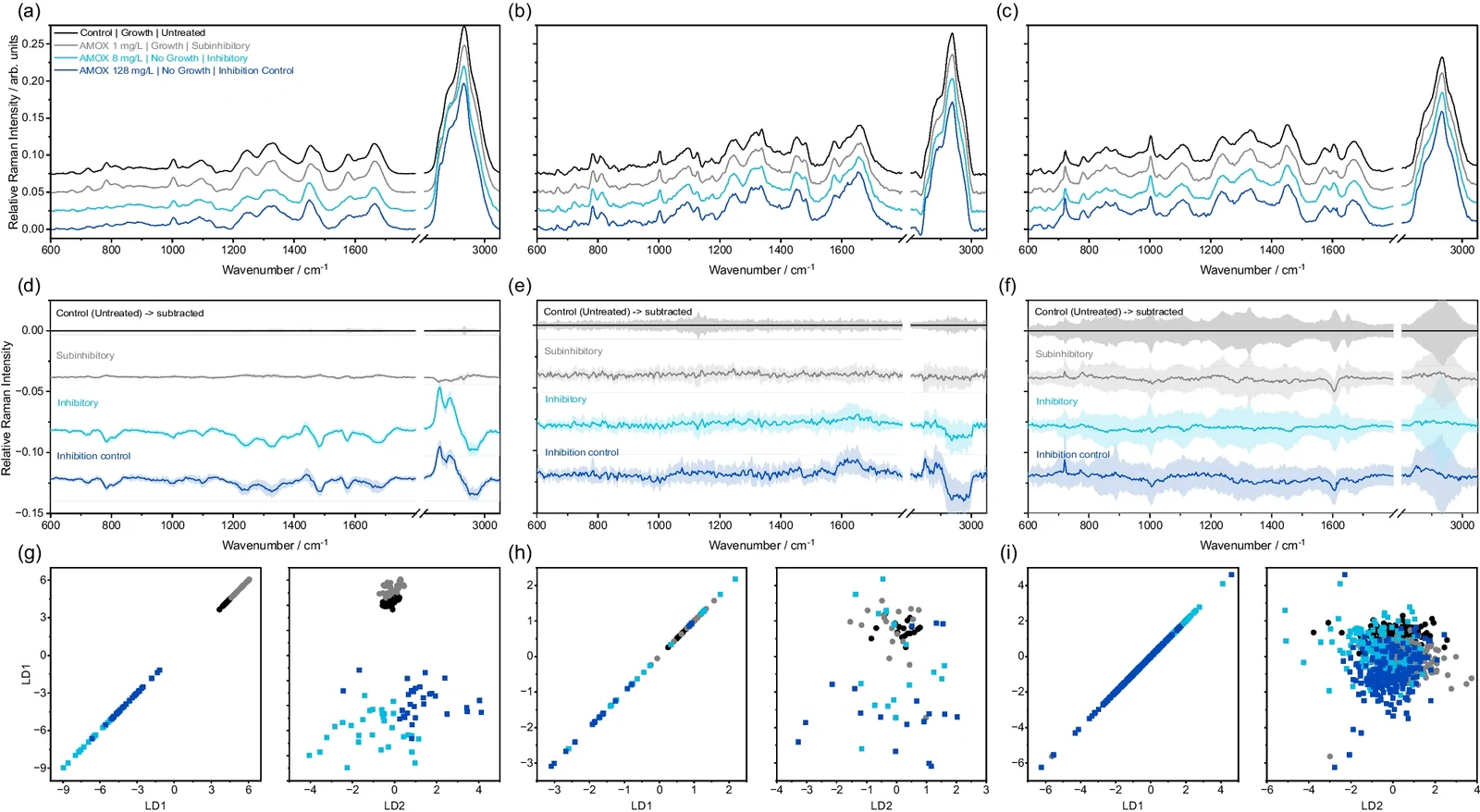
Results of the data analysis for each of the three measurement procedures investigated for amoxicillin: averaged spectra for the **a** dry-bulk measurements, **b** DEP-chip measurements, **c** single-cell measurements; difference spectra generated by subtracting the untreated control from treatment spectra for the **d** dry-bulk measurements, **e** DEP-chip measurements, **f** single-cell measurements (for all the above spectra offset was introduced for better visualization); LDA linear discriminant plots for the PCA-LDA Leave one Batch-out cross validation (LOBOCV) analysis model for the **g** dry-bulk measurements, **h** DEP-chip measurements, **i** single-cell measurements.

For the dry-bulk measurements on CaF_2_, clear spectral differences between “growth” and “no growth” conditions were observed (Fig. 3a, d). Difference spectra revealed minimal changes between untreated and sub-inhibitory samples, while inhibitory conditions showed pronounced spectral alterations. This resulted in well-separated clusters and high classification accuracy (Fig. 3g), highlighting the strong performance of Raman-based AST within 90 min of antibiotic exposure. The high reproducibility of this approach is further reflected in the low spectral variance across replicate measurements. However, this method requires centrifugation and washing steps, which may introduce additional stress to the cells and potentially amplify observed spectral differences [15].

Measurements using the DEP-chip platform also enabled reliable discrimination between growth and inhibition states while allowing analysis in a closed liquid system without additional washing steps. The formation of bacterial clusters (“clouds”) resulted in reproducible spectra, comparable to bulk measurements. However, a slight decrease in class separation was observed, particularly for the “no growth” conditions (Fig. 3h), indicating increased variability compared to dry-bulk measurements.

In contrast, single-cell measurements on the filtration substrate exhibited substantially higher spectral variability and reduced classification performance (Fig. 3f, i). This can be attributed to the inherent heterogeneity of individual cells and the fact that each measurement captures only a single bacterium. In contrast to bulk or clustered measurements, where signals are averaged over multiple cells, single-cell spectra are more sensitive to variations in physiological state and antibiotic response. Consequently, a larger number of measurements is required to achieve statistically robust classification.

In addition to biological variability, differences in measurement configuration contribute to the observed performance. Bulk and DEP-based approaches effectively probe multiple cells within the confocal volume, leading to signal averaging and improved reproducibility. In contrast, single-cell measurements are more susceptible to positioning and focusing inaccuracies, which can further increase spectral variability.

Overall, while all three approaches enable Raman-based detection of antibiotic-induced phenotypic changes, their performance differs substantially. Dry-bulk measurements provide the highest robustness but require hands-on sample preparation. DEP-based measurements offer a favourable compromise between performance and experimental complexity in a closed system. Single-cell measurements, while advantageous for low cell concentrations, currently suffer from reduced robustness due to increased biological and technical variability.

### Levofloxacin

Levofloxacin, a fluoroquinolone antibiotic, inhibits bacterial DNA replication by targeting DNA gyrase and topoisomerase IV [16]. In contrast to β-lactam antibiotics such as amoxicillin, which directly disrupt cell wall synthesis and rapidly induce cell lysis, levofloxacin primarily interferes with DNA processing and cell division. Consequently, the phenotypic response to levofloxacin may include heterogeneous and transient states, including phenomena associated with persistence, as described for bacteria treated with fluoroquinolones [17]. Such heterogeneous responses are a common feature of populations exposed to antibiotics [4, 18] and may be more evident under experimental conditions where growth inhibition is incomplete, such as at concentrations close to the minimum inhibitory concentration (MIC).

We therefore expect Raman-based discrimination between growth and inhibition in general to be less pronounced or more variable compared to amoxicillin, especially under sub-inhibitory conditions.

Growth assessment by BMD tests (see Figure SI 3.1b) confirmed that untreated samples were growing, while inhibitory and inhibition-control concentrations showed no growth. In contrast, the sub-inhibitory concentration (0.125 mg/l) exhibited inconsistent behaviour across the individual replicates: in most replicates, growth was inhibited, but in some, growth was delayed (see Figure SI 3.2).

**Fig. 4:**
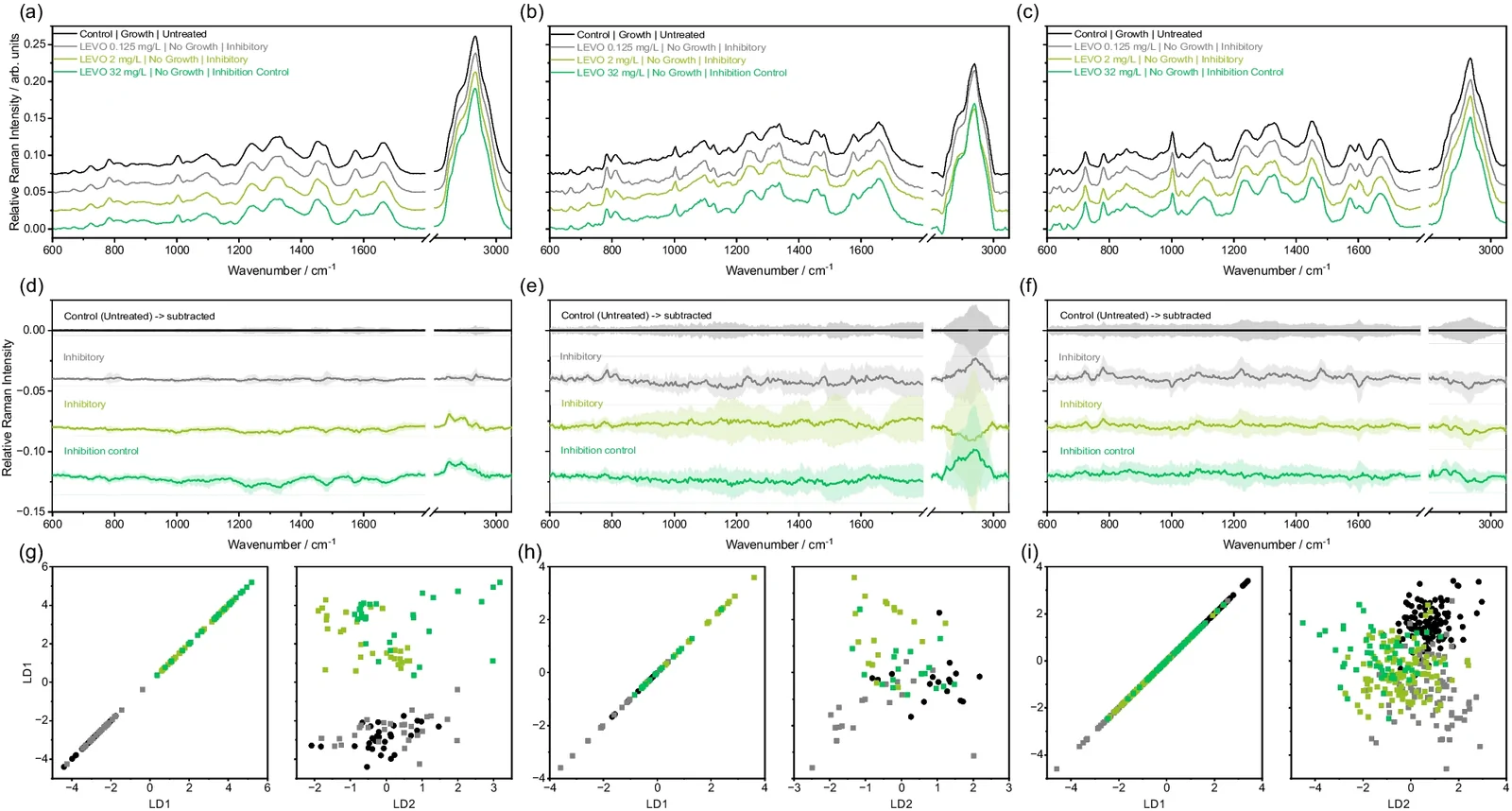
Results of the data analysis for each of the three measurement procedures investigated for levofloxacin: averaged spectra for the **a** dry-bulk measurements, **b** DEP-chip measurements, **c** single-cell measurements; difference spectra generated by subtracting the untreated control from treatment spectra for the **d** dry-bulk measurements, **e** DEP-chip measurements, **f** single-cell measurements (for all the above spectra offset was introduced for better visualization); LDA linear discriminant plots for the PCA-LDA Leave one Batch-out cross validation (LOBOCV) analysis model for the **g** dry-bulk measurements, **h** DEP-chip measurements **i** single-cell measurements.

For dry-bulk Raman measurements on CaF_2_ substrates, distinct spectral differences were observed between the untreated control and the inhibitory conditions, resulting in well-separated clusters and high classification performance (Fig. 4g). Difference spectra confirmed pronounced biochemical changes at higher antibiotic concentrations (Fig. 4d). Notably, the sub-inhibitory concentration was classified as “grown” by the Raman-based model, in contrast to its assignment based on OD600 measurements. This behaviour can be explained by the proximity of this concentration to the MIC: while short-term phenotypic changes within the 90 min incubation period remain limited, longer incubation allows the antibiotic effect to manifest more clearly. Thus, Raman spectroscopy appears to capture early-stage or transient phenotypic states that are not yet reflected in bulk growth measurements.

Measurements using the DEP-chip platform showed the same basic trend, with the untreated control and sub-inhibitory concentration clustering closer together, while higher antibiotic concentrations formed a separate group (Fig. 4h). However, compared to dry-bulk measurements, increased overlap between clusters was observed, indicating reduced classification robustness. As discussed for amoxicillin, this can be attributed to increased variability in the measurement conditions and the absence of signal averaging over fully immobilized cell populations.

Single-cell measurements on the filtration substrate also exhibited a higher spectral variability compared to the dry bulk samples and yielded lower classification performance (Fig. 4i). While a general separation between untreated and antibiotic-treated samples was still observable, there was some cluster overlap. This is consistent with the expected heterogeneity of individual bacterial responses to fluoroquinolone treatment, which affects DNA replication and cell division in a less uniform manner than cell wall-targeting antibiotics. As a result, single-cell measurements are particularly sensitive to differences in the physiological state of individual cells and require larger datasets for robust classification.

Overall, Raman spectroscopy successfully detected antibiotic-induced phenotypic changes for levofloxacin across all measurement platforms. However, compared to amoxicillin, classification performance was reduced, except for the filtration-based approach, where the results for levofloxacin were improved. This highlights the influence of the mode of action of antibiotics on the sample preparation performance as well as the Raman measurements. Among the investigated approaches, dry-bulk measurements provided the highest robustness, while DEP-based measurements offered a promising compromise between performance and experimental complexity in a closed system. Single-cell measurements, although advantageous for low cell concentrations, can be limited by increased biological and technical variability, but the performance depends on the class of the antibiotic.

### Comparison

Table 1 summarizes the classification performance of the Raman-based AST approach for amoxicillin and levofloxacin for the three investigated measurement strategies. Globally, the results demonstrate that Raman spectroscopy enables reliable discrimination between bacterial growth and inhibition within short incubation times. However, the classification performance depends on both the antibiotic mechanism of action and the chosen measurement configuration.

**Table 1:** PCA-LDA model for predicting the two classes “growth” and “no growth” at spectral and batch level when treated with the investigated antibiotics.

		Amoxicillin			Levofloxacin		
		Dried bulk					
**Spectral level**		**Predicted**		**Balanced** **accuracy**	**Predicted**		**Balanced** **accuracy**
		No Growth	Growth		No Growth	Growth	
True	No Growth	60	0	**100%**	70	18	**81.4%**
	Growth	0	60		5	25	
**Batch level**		**Predicted**		**Balanced** **accuracy**	**Predicted**		**Balanced** **accuracy**
		No Growth	Growth		No Growth	Growth	
True	No Growth	3	0	**100%**	3	0	**100%**
	Growth	0	3		0	3	
		**DEP-Chip**					
**Spectral level**		**Predicted**		**Balanced** **accuracy**	**Predicted**		**Balanced** **accuracy**
		No Growth	Growth		No Growth	Growth	
True	No Growth	22	14	**76.4%**	21	33	**63.9%**
	Growth	3	33		2	16	
**Batch level**		**Predicted**		**Balanced** **accuracy**	**Predicted**		**Balanced** **accuracy**
		No Growth	Growth		No Growth	Growth	
True	No Growth	2	1	**83.3%**	1	2	**66.7%**
	Growth	0	3		0	3	
		**Single-cell**					
**Spectral level**		**Predicted**		**Balanced** **accuracy**	**Predicted**		**Balanced** **accuracy**
		No Growth	Growth		No Growth	Growth	
True	No Growth	216	151	**64.5%**	168	75	**79.5%**
	Growth	101	238		11	97	
**Batch level**		**Predicted**		**Balanced** **accuracy**	**Predicted**		**Balanced** **accuracy**
		No Growth	Growth		No Growth	Growth	
True	No Growth	2	1	**66.7%**	2	1	**83.3%**
	Growth	1	2		0	3	

For amoxicillin, separation between “growth” and “no growth” conditions was achieved across all platforms, with the highest accuracy observed for dried bulk measurements. This can be attributed to the mode of action of β-lactam antibiotics, which rapidly disrupt cell wall synthesis and induce pronounced and relatively uniform phenotypic changes across the bacterial population. In contrast, levofloxacin exhibited slightly reduced classification performance, particularly near the minimum inhibitory concentration (MIC). As a fluoroquinolone targeting DNA replication, levofloxacin induces more gradual and heterogeneous cellular responses, which are less consistently captured within the short incubation time. This difference was most evident for sub-inhibitory conditions, where Raman-based classification and growth-based reference methods diverged, reflecting transient or delayed phenotypic effects. Interestingly, for the filtration-based single-cell approach the classification results for levofloxacin and amoxicillin were reversed. Here the results for levofloxacin were clearly superior. As amoxicillin induces cell lysis, it is plausible that the sample preparation via filtration introduces a bias towards selectively enriching cells that are still intact. The cells already strongly affected by the antibiotic might pass through the pores of the filter more easily and are no longer available for measurements.

Across both antibiotics, dried bulk measurements on CaF_2_ substrates clearly provided the highest robustness and classification accuracy. This is attributed to signal averaging over large bacterial populations and high spectral reproducibility. However, this approach requires centrifugation and washing steps and is limited to open measurement configurations, which restricts its applicability in clinical settings.

Measurements using the DEP-chip platform represent a promising alternative, enabling Raman analysis in a closed liquid system without extensive sample preprocessing. While classification performance was reduced compared to dried bulk measurements, the approach maintained acceptable discrimination between growth states for amoxicillin. This reduction in performance is likely due to increased variability in the measurement environment, as well as to the dependence on efficient cell separation for the formation of bacterial clusters. Further experiments using an excitation wavelength of 785 nm (see SI7) show that the excitation wavelength can influence spectral quality and classification performance, which may be due to lower fluorescence background and improved signal stability under these conditions.

Single-cell measurements on filtration substrates showed the lowest classification performance for amoxicillin, but very promising results for levofloxacin. This is an example underlining the critical importance of carefully considering the effect of sample preparation on the cells. We suspect that the cell lysis induced by amoxicillin at inhibitory concentrations might have caused the cells affected the most to pass through the filter and evade measurements. While this approach enables measurements at low bacterial concentrations and offers high flexibility in sample handling, it is inherently more sensitive to biological heterogeneity and technical variability.

Beyond classification performance, practical aspects of sample preparation and especially measurement time are critical for the implementation of Raman-based AST in clinical workflows. Based on the experimental workflows, the overall assay time can be divided into three main components: antibiotic incubation (90 min - identical across all approaches), sample preparation, and Raman data acquisition.

As summarized in Table SI 4.2, clear differences between the investigated measurement strategies are observed. Dried bulk measurements require approximately 40 min of sample preparation, mainly due to centrifugation, washing, and drying steps. The subsequent Raman acquisition is relatively fast, resulting in total assay times of approximately 3 h for both antibiotics. This makes the dried bulk approach efficient in terms of measurement time, albeit at the cost of more labour-intensive sample preparation.

DEP-based measurements show the shortest sample preparation time (~ 25 min), as no extensive washing steps are required and measurements can be performed directly in a liquid environment. Combined with moderate acquisition times, this results in the shortest overall assay duration (approximately 2 h 25 min to 2 h 35 min). Notably, the use of a 785 nm excitation source further reduced acquisition times significantly (2 h 7 min), highlighting the strong influence of the measurement setup on overall performance. These results underline the potential of DEP-based approaches for time-efficient and clinically compatible Raman-AST workflows.

In contrast, single-cell measurements exhibit substantially longer acquisition times, as a large number of individual spectra must be recorded to achieve statistically robust classification. Despite similar or longer sample preparation times (~ 60 min), the total assay duration increases to approximately 6–8 h, making this approach currently less suitable for rapid diagnostic applications.

Overall, these findings demonstrate that the total analysis time is not solely determined by sample preparation, but strongly influenced by the measurement strategy and instrumental configuration. While dried bulk measurements offer high robustness and relatively short acquisition times, DEP-based approaches provide the most favourable balance between speed, experimental complexity, and compatibility with closed systems. In contrast, single-cell measurements remain limited by extended acquisition times, despite their advantages for low cell concentrations.

Taken together, the results highlight that Raman-based AST performance is governed by an interplay between antibiotic mechanism, sample presentation, and measurement configuration. While dried bulk measurements provide the highest analytical robustness, DEP-based approaches offer a compelling compromise between performance, speed and clinical compatibility. The reduced performance observed for levofloxacin, particularly under sub-inhibitory conditions, further underscores the importance of considering antibiotic-specific response dynamics when designing and evaluating Raman-based AST strategies.

## Conclusion

In this study, we systematically compared three Raman-based measurement strategies - dried bulk measurements, DEP-assisted measurements in liquid, and single-cell measurements on filtration substrates - for rapid phenotypic antibiotic susceptibility testing (AST).

Across all investigated approaches, dried bulk measurements clearly provided the highest classification robustness due to signal averaging over large bacterial populations, albeit at the cost of additional sample preparation steps. DEP-based measurements in liquid represent a promising compromise, enabling analysis in a closed and potentially clinically compatible system while maintaining good predictive performance and speed. Single-cell measurements offer advantages for low bacterial concentrations and minimal pre-processing, but are currently limited by increased biological and technical variability and long data acquisition times.

Some limitations of the present study should be considered. The investigation was restricted to a single Gram-negative model organism (*Escherichia coli* ATCC 25922) and one representative antibiotic per class. While the observed trends provide valuable mechanistic insights, their general validity must be confirmed by extending the analysis to a broader range of bacterial species, including Gram-positive organisms, as well as additional antibiotics with diverse modes of action. Such studies will be essential to assess the robustness and generalizability of Raman-based AST across clinically relevant scenarios.

Overall, this work highlights that the successful implementation of Raman-based AST requires a careful balance between measurement robustness, sample preparation complexity, and clinical applicability. By systematically comparing different measurement strategies and linking their performance to antibiotic mode of action, this study provides a framework for the rational development of Raman-based diagnostic platforms. Future efforts should focus on expanding biological diversity, optimizing measurement conditions, and integrating sample preparation and detection into streamlined, clinically compatible workflows.

## Supplementary Information

Below is the link to the electronic supplementary material.

ESM 1 Supplementary Material 1 (PDF 7.15 MB)

## Data Availability

The raw data are available on request by contacting the corresponding author.
