# Supplementary material for: From bulk samples to single cells: measurement strategies in Raman-based antibiotic susceptibility testing
Source: Anal Bioanal Chem. 2026 Jun 3;418(23):7913–25. doi: 10.1007/s00216-026-06558-3 (PMC13744562; doi:10.1007/s00216-026-06558-3)
Supplement: Supplementary file 1 [file 216_2026_6558_MOESM1_ESM.pdf]

From Bulk Samples to Single Cells: Measurement Strategies in Raman-Based Antibiotic Susceptibility Testing

Supporting information

Marie-Luise Enghardt [1,2,3]*, Andreea Winterfeld [1]*, Susanne Pahlow [1], Anja Silge [1,3], Jürgen Popp [1,2,3]**

[1] Leibniz Institute of Photonic Technology, Member of Leibniz Health Technologies, Member of the Leibniz Centre for Photonics in Infection Research (LPI), Jena Germany

[2] Institute of Physical Chemistry (IPC) and Abbe Center of Photonics (ACP), Friedrich Schiller University Jena, Member of the Leibniz Centre for Photonics in Infection Research (LPI), Helmholtzweg 4, 07743 Jena, Germany

[3] InfectoGnostics Research Campus Jena, Center of Applied Research, Philosophenweg 7, 07743 Jena, Germany

***** Contributed equally

****** juergen.popp@leibniz-ipht.de; +49 3641 206300; JP

Contents

1. Correlation between optical density (OD_{600}) and viable cell count (CFU/mL) 2
2. Determination of antibiotic concentrations in AT2 50 % medium 3
3. Validation via Broth microdilution test 5
4. Total number of spectra/samples and measurement time used within the different measurement approaches 7
5. Estimation of the effective illumination spot size diameter and depth of field for the Raman measurements 9
6. PCA-loadings of the Raman spectra used for class separation 11
7. Resistance testing of Levofloxacin on the DEP-Chip with 785 nm 12

1. Correlation between optical density (OD_{600}) and viable cell count (CFU/mL)

To determine the relationship between optical density and the number of viable cells, the exponential growth phase was first identified using the OD_{600} growth curve (Figure SI 1.1a). For each time point, serial dilutions in PBS were prepared individually so as to achieve a countable number of colonies. Aliquots of 100 µL of the corresponding dilution were plated on LB agar and incubated at 37 °C for 24 hours (Table SI 1.1). Plate counts corresponding to the exponential growth phase were selected and correlated with the respective OD_{600} values (Figure SI 1.1b).

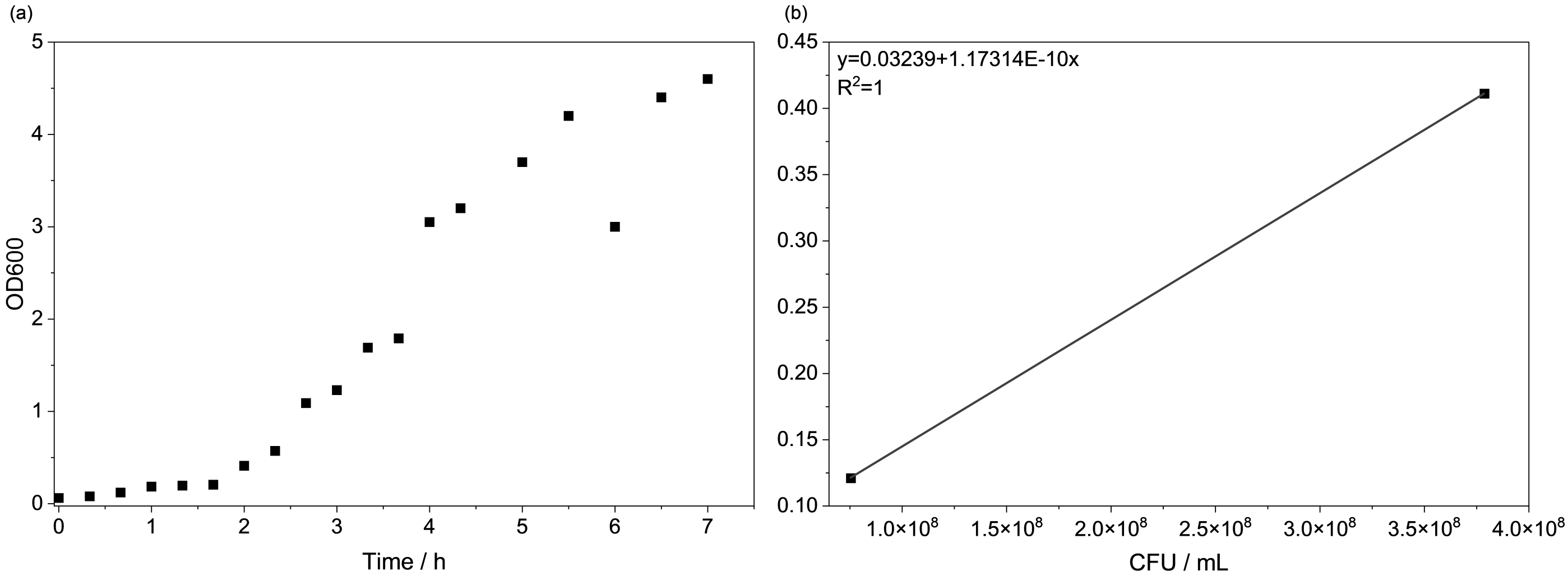

Figure SI 1.1 Correlation between OD_{600} and CFU/mL for *E. coli* ATCC 25922. (a) Growth curve used to identify the exponential phase. (b) Correlation between OD_{600} and CFU/mL based on selected data points from this phase.

The conversion was derived using a linear approximation, resulting in ~3.27×10^7 CFU/mL at OD_{600} = 0.08.

Table SI 1.1 Colony counts, applied dilution factors, and calculated CFU/mL values used for OD_{600}-CFU/mL correlation.

Time [min]	OD600	Dilution	Plate a	Plate b	Plate c	Mean CFU	CFU/mL	Mean CFU/mL
0	0.062	1:5.000	206	236	222	221.3	1.11×10^7	1.24×10^7
		1:50.000	25	31	26	27.3	1.37×10^7	
40	0.121	1:40.000	238	207	228	224.3	8.97×10^7	7.55×10^7
		1:400.000	19	16	11	15.3	6.13×10^7	
120	0.411	1:40.000	691*	687*	623*	667	2.67×10^8	3.79×10^8
		1:400.000	78	110	96	94.7	3.79×10^8	
240	3.05	1:100.000	205	213	215	211	2.11×10^8	2.12×10^8
		1:1.000.000	18	22	24	21.3	2.13×10^8	

*Colony counts that exceeded the countable range (>300 colonies per plate) were excluded from CFU calculation and averaging.

2. Determination of antibiotic concentrations in AT2 50 % medium

As the Raman measurements were carried out in AT2 50 % medium using an inoculum that differed from the standardized EUCAST conditions with Mueller-Hinton medium and an inoculum of 5×10⁵ CFU/mL [1], the antibiotic concentrations for *E. coli* ATCC 25922 were determined experimentally using broth microdilution (Figure SI 2.1).

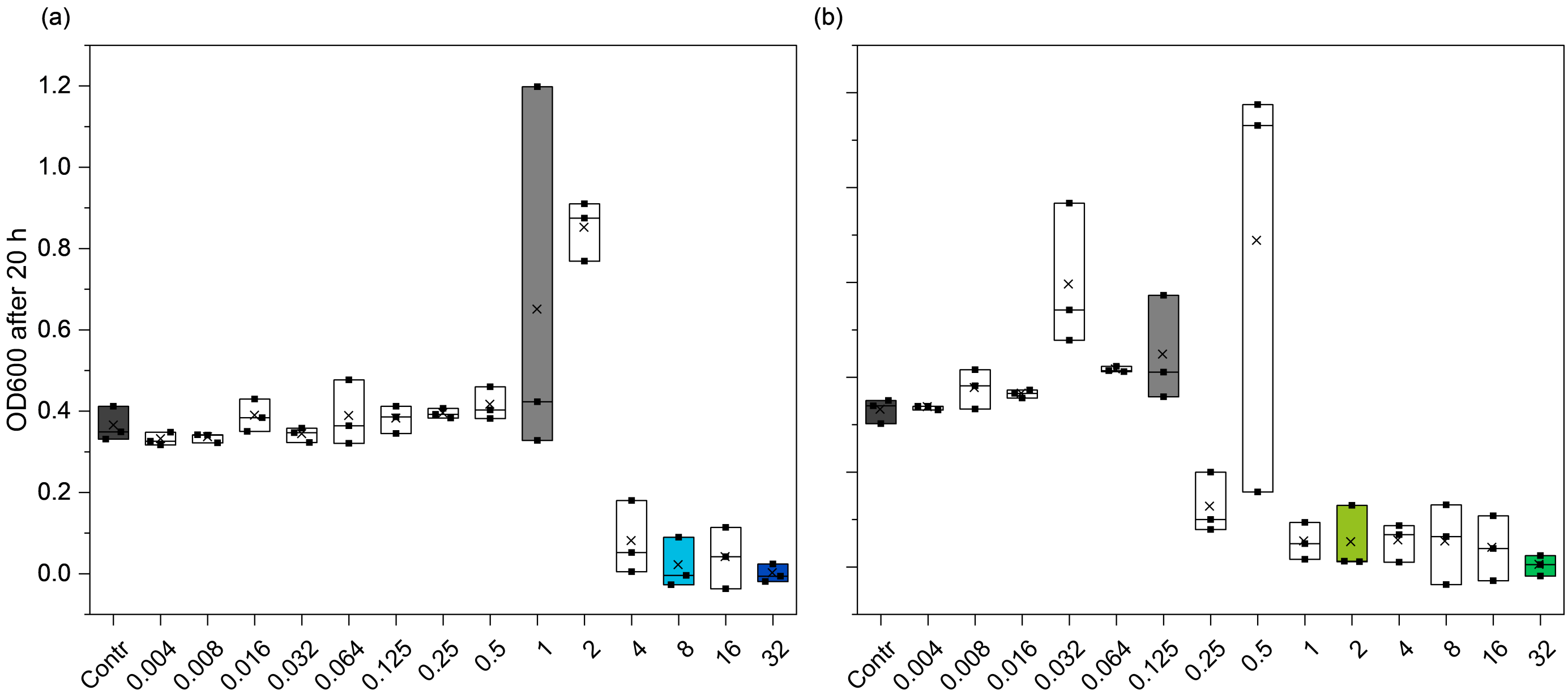

Figure SI 2.1 Endpoint OD_{600} measurements after 20 h incubation of *E. coli* ATCC 25922 in AT2 50 % medium at different concentrations of (a) amoxicillin and (b) levofloxacin. The boxes represent the interquartile range (25th to 75th percentile), the centre line indicates the median, the x represents the mean value, and the individual points represent individual measurements. Coloured concentrations indicate the selected conditions used for the Raman experiments; for amoxicillin, 32 mg/L is shown as a representative inhibitory concentration corresponding to 128 mg/L.

The experimentally determined MIC values varied depending on the antibiotic (Table SI 2.1). For amoxicillin, a clear MIC was observed at 4 mg/L, which is consistent with the EUCAST reference values. In contrast, growth inhibition was observed for levofloxacin over a broader concentration range at 0.25–1 mg/L, indicating a deviation from the EUCAST reference values (0.016–0.03 mg/L). Based on these results, four concentrations were selected for the Raman experiments: an untreated control, a sub-inhibitory concentration (one dilution step below the MIC) and two inhibitory concentrations (one and five dilution steps above the MIC, respectively). This selection ensured that different phenotypic states were covered, ranging from unrestricted growth to severe inhibition.

Table SI 2.1 Comparison of EUCAST reference MIC values and experimentally determined MIC values in AT2 50 % medium for *E. coli* ATCC 25922, and corresponding antibiotic concentrations selected for Raman measurements.

Antibiotic	EUCAST-MIC [mg/L] 10^5 CFU/mL in MH	RBA-MIC [mg/L] 3×10^7 CFU/mL in AT2 50 %	RBA-concentrations			
			untreated control	MIC-1 sub-inhibitory	MIC+1 inhibitory	MIC+5 inhibition control
Amoxicillin	2 – 4	4	0	1	8	128
Levofloxacin	0.016 – 0.03	0.25-1	0	0.125	2	32

3. Validation via Broth microdilution test

The OD_{600} endpoint measurements shown in Figure SI 3.1 comprise data from several experimental batches and serve as a reference (gold standard) for validation based on the broth microdilution method. However, for the first batch, no endpoint measurements were available after 20 hours. To ensure a consistent dataset and to validate the classifications, three additional biological replicates were performed under identical conditions and included in the analysis. The measurements suggest that, under the given conditions in the AT2 50 % medium, 0.125 mg/L is close to the effective MIC, leading to a heterogeneous growth response across the replicates. To further illustrate this variability, the corresponding OD_{600} growth curves are shown in Figure SI 3.2. The combined data from all batches show that some of the replicates exhibit delayed regrowth, which explains the increased variability in the endpoint measurements and the partial deviation from the classification derived from the broth microdilution reference.

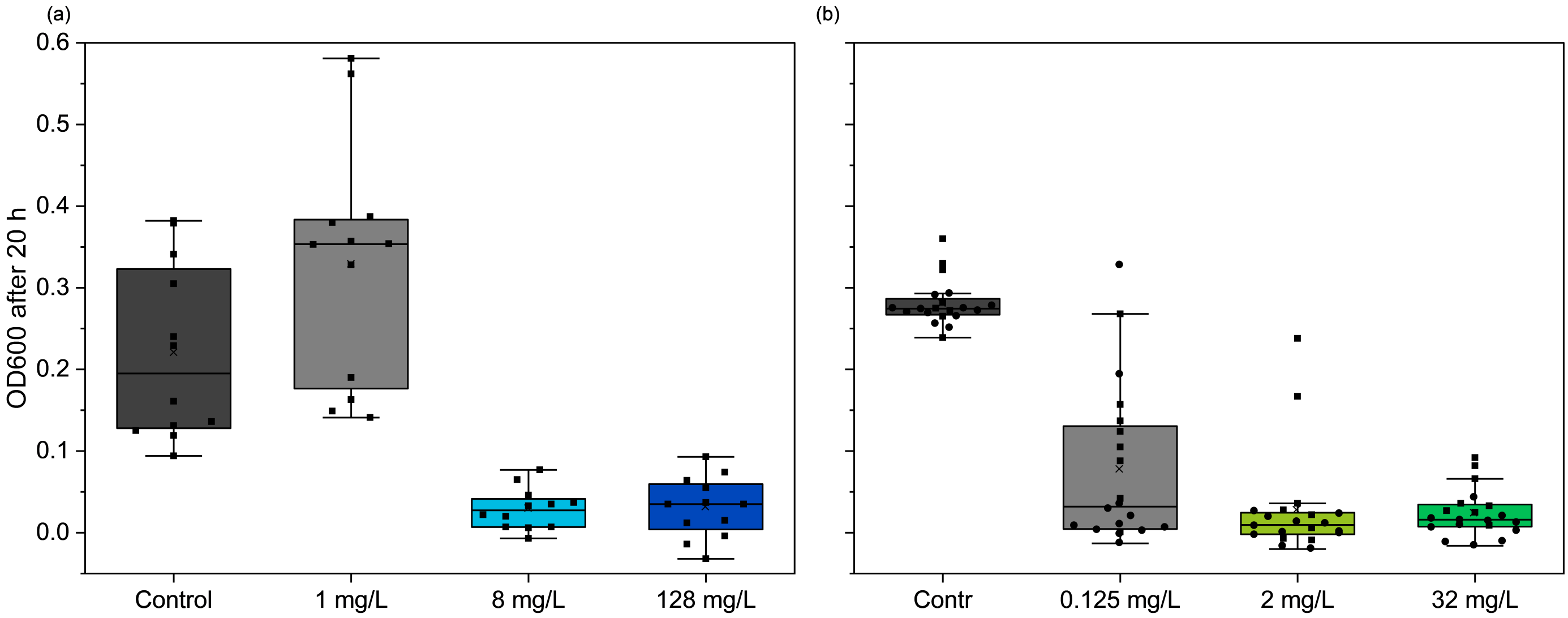

Figure SI 3.1 Endpoint OD_{600} measurements performed after 20 h interaction time for the same samples that were measured within the main Raman experiments. *E. coli* ATCC 25922 and investigated concentrations of (a) amoxicillin and (b) levofloxacin. The boxes represent the interquartile range (25th to 75th percentile), the centre line indicates the median, the x represents the mean value, and the individual points represent individual measurements. For levofloxacin, the validation data for batches 2 and 3 are represented as squares, while the three batches of repeat measurements are represented as circles.

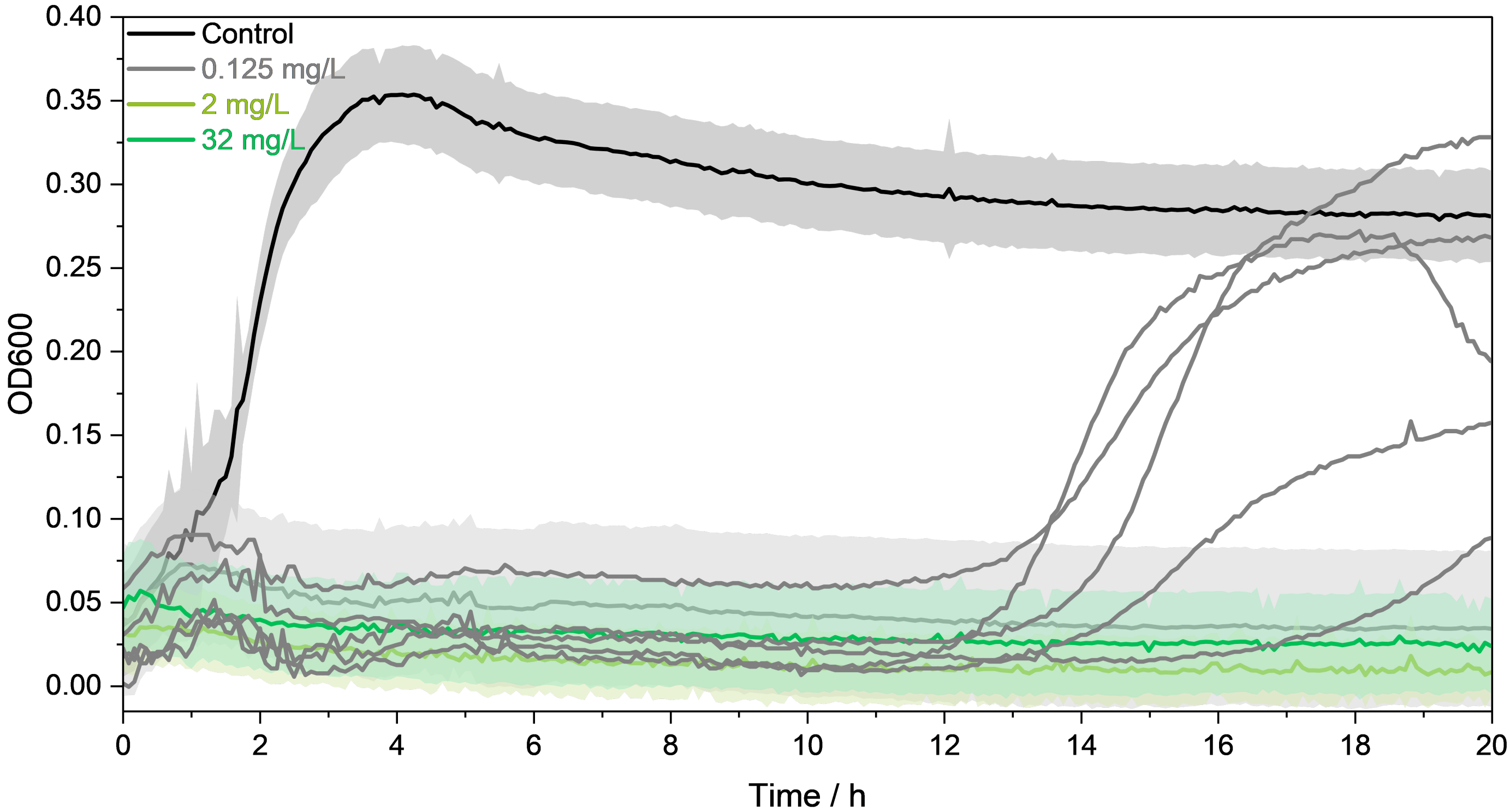

Figure SI 3.2 Combined OD600 growth curves for *E. coli* ATCC 25922 at different levofloxacin concentrations across all three batches. The solid lines represent the mean of all biological replicates per concentration, and the hatched areas indicate the corresponding standard deviation. For 0.125 mg/L levofloxacin, individual outlier isolates are also shown. Of a total of 20 replicates, five exhibited delayed regrowth and are therefore shown separately.

To investigate the observed variability, additional broth microdilution experiments were performed under identical AT2 50 % conditions. Across these repeated measurements, the effective MIC for levofloxacin was consistently determined to be 0.125 mg/L. This confirms that the heterogeneous growth behaviour observed at this concentration reflects its proximity to the inhibitory threshold.

4. Total number of spectra/samples and measurement time used within the different measurement approaches

The size of the datasets varies considerably between the different measurement approaches: single-cell measurements require a significantly higher number of spectra to achieve a robust statistical representation, whereas approaches using dried sample material and DEP chips require fewer, measurements per experimental condition.

Table SI 4.1 Total number of Raman spectra acquired for each measurement approach, antibiotic, and treatment condition included in the data analysis summarized for respectively three biological replicates.

Antibiotic	Method	Raman system and excitation	Untreated control	(Sub)-inhibitory	Inhibitory	Inhibition control	Total
Amoxicillin	Dried Bulk	WITec alpha300R 514 nm	30	30	30	30	120
	DEP-Chip	WITec alpha300 Ri 532 nm	18	18	18	18	72
	Single-Cell	Bio Particle Explorer 532 nm	154	180	152	129	615
Levofloxacin	Dried Bulk	WITec alpha300R 514 nm	30	30	30	30	120
	DEP-Chip	WITec alpha300 Ri 532 nm	18	18	18	18	72
	DEP-Chip	WITec alpha300 Ri 785 nm	12*	12*	12*	12*	48*
	Single-Cell	Bio Particle Explorer 532 nm	160	179	169	198	706

Table SI 4.2 Total measurement time of Raman spectra acquired for each measurement approach, laser wavelength and antibiotic for each biological replicate.

Antibiotic	Method	Raman system and excitation	Sample prepera-tion time*	Biological replicate 1	Biological replicate 2	Biological replicate 3	Mean with Standard Deviation	Total time of AST*****
Amoxicillin	Dried Bulk	WITec alpha300R 514 nm	40 min**	1 h 19 min	0 h 37 min	0 h 31 min	0 h 49 min ± 22 min	2 h 59 min
	DEP-Chip	WITec alpha300 Ri 532 nm	25 min***	0 h 55 min	0 h 35 min	0 h 28 min	0 h 40 min ± 11 min	2 h 35 min
	Single-Cell	Bio Particle Explorer 532 nm	60 min****	7 h 30 min	4 h 10 min	4 h 38 min	5 h 26 min ± 1 h 48 min	7 h 56 min
Levofloxacin	Dried Bulk	WITec alpha300R 514 nm	40 min**	0 h 45 min	1 h 2 min	0 h 55 min	0 h 54 min ± 7 min	3 h 4 min
	DEP-Chip	WITec alpha300 Ri 532 nm	25 min***	0 h 43 min	0 h 25 min	0 h 23 min	0 h 30 min ± 9 min	2 h 25 min
	DEP-Chip	WITec alpha300 Ri 785 nm		-	0 h 12 min	0 h 13 min	0 h 12 min ± 0.5 min	2 h 07 min
	Single-Cell	Bio Particle Explorer 532 nm	60 min****	3 h 31 min	3 h 55 min	3 h 41 min	3 h 42 min ± 12 min	6 h 12 min

* estimated retrospectively based on the experimental procedure, as the preparation time was not specifically recorded

** centrifugation and washing steps requiring 20 minutes, and a drying time of ~20 minutes prior to the measurement

*** 15 min for OD adjustment with chip loading and ~10 min DEP enrichment prior to the measurement

**** centrifugation and washing steps requiring 20 minutes, 20 min OD adjustment and filtration and a drying time of ~20 minutes prior to the measurement

***** composed of 90 minutes interaction time with the antibiotic, followed by sample preparation and the average measurement time

5. Estimation of the effective illumination spot size diameter and depth of field for the Raman measurements

The Raman measurements introduced here were performed on three different systems, using three different microscope objectives. To compare and discuss these results, we investigated the details of the measurement systems and estimated the illumination effective spot size diameter and depth of field for each of them. The beam diameter was measured for the two different WITec systems using a Thorlabs Shack-Hartmann Wavefront Sensor, Model: WFS40-5C [2]. The values are given in Table SI 5.1.

Table SI 5.1 Summary of the system parameters and estimated the effective spot size diameter and depth of field.

Raman system	Microscope Objective						D_{laser} (4)	$N.A._{eff}$ (5)	M^2 (6)	λ (7)	Measurement spot size		
	producer	Mag.	N.A.	f_{TL} (1)	f_{Obj} (2)	D_{pupil} (3)					r_{spot}	d_{spot}	DoF (8)
		×		mm	mm	mm	mm			nm	µm	µm	µm
WITec alpha300R	Zeiss	20	0.40	165	8.25	6.60	2.50 *	0.15	1.1	514.8	1.08	2.16	14.26
WITec alpha300 Ri	Nikon	60	0.85	200	3.33	5.67	2.10 *	0.32	1	532.053	0.54	1.08	3.42
Bio Particle Explorer	Olympus	100	0.90	180	1.8	3.24	1.20 **	0.33	1	532	0.51	1.02	3.05

(1) Design tube lens focal length according to datasheets

(2) Microscope objective focal length

(3) Microscope objective pupil diameter

(4) Laser beam diameter at the microscope objective entrance pupil

* Measured with the Shack-Hartmann sensor

** Data available in the laser datasheet

(5) Effective N.A. due to underfilling the microscope objective entrance pupil

(6) Laser beam quality factor – the case of ideal Gaussian profile

(7) Measured excitation wavelength

(8) Depth of field

Table SI 5.2 The Beam View panel of the Shack-Hartmann sensor: overview of the intensity distribution within the active camera sensor area. This display is comparable to that of a camera-based Beam Profiler, but with the constraint of a lower resolution.

WITec upright - WITec alpha300R Raman	WITec inverse - WITec alpha300 Ri Raman	Bio Particle Explorer, rap.ID
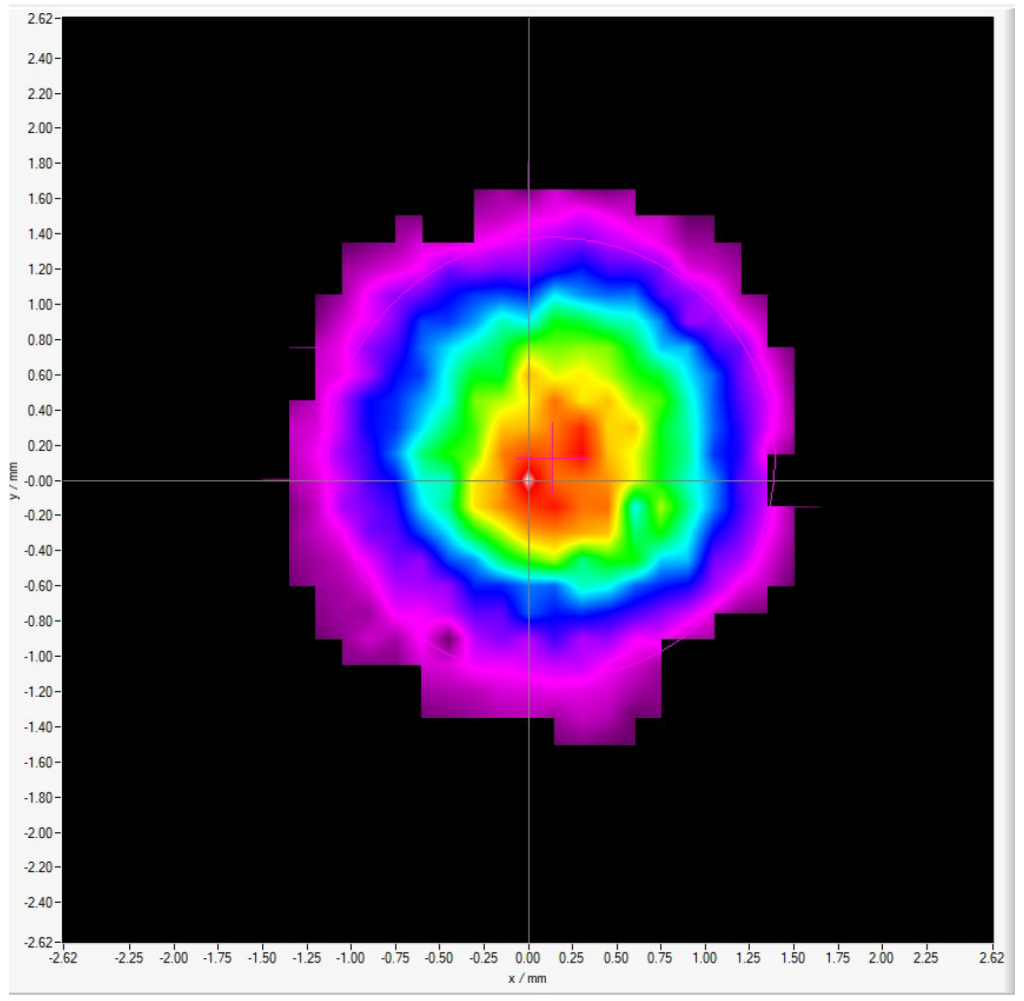	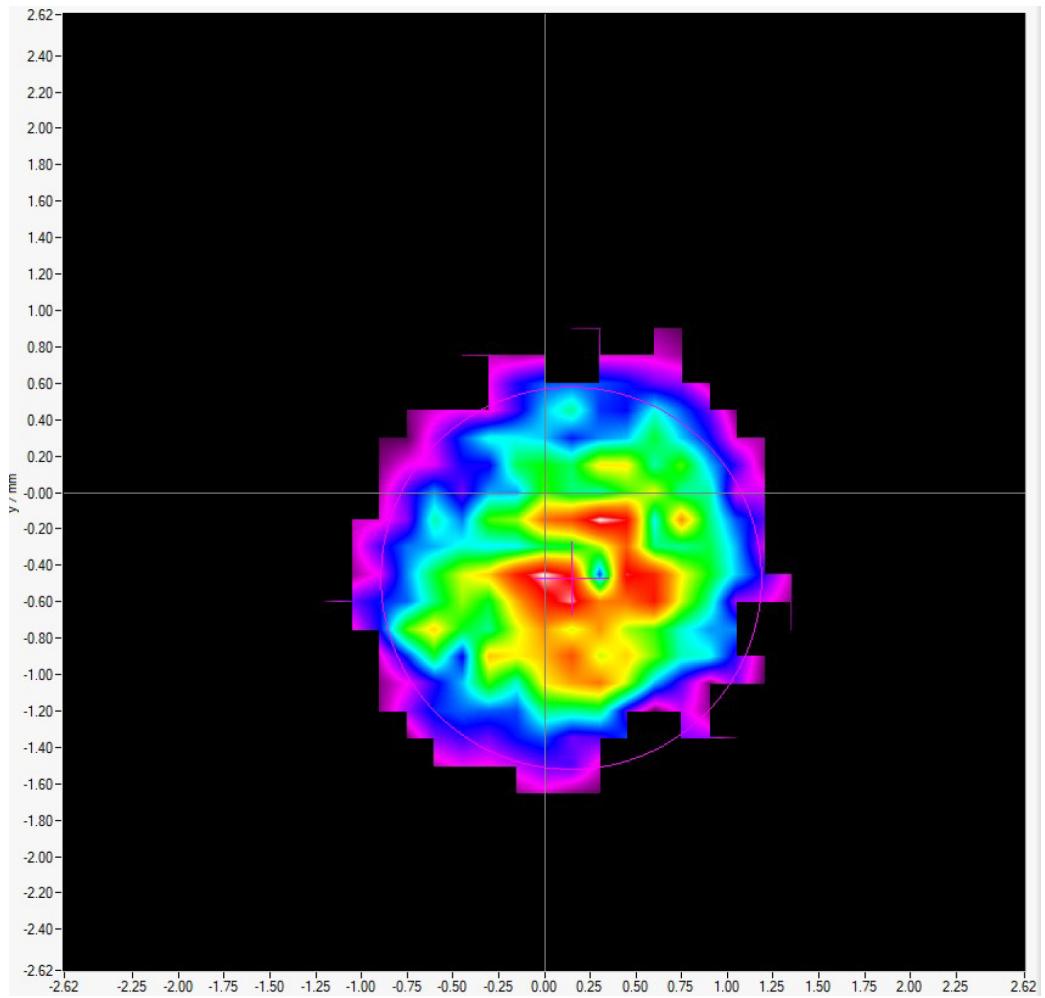	Due to instrument design, no direct access to the collimated laser beam entering the microscope objective is given. The value of the laser beam diameter is provided in the datasheet of the laser.

Microscope objective focal length: $f_{Obj} = \frac{f_{TL}}{Mag.}$

Entrance lens/pupil diameter (also known as the entrance aperture diameter) represents the effective beam diameter required for the objective to operate correctly: $D_{pupil} = 2 * NA * \ f_{Obj}$

The effective numerical aperture of an objective lens does not necessarily match its nominal (labeled) value. Instead, it depends on the actual diameter of the light beam that fills the objective entrance pupil: $NA_{eff} = NA_{objectiv} * \frac{D_{laser\ beam}}{D_{pupil}}$

Laser focus spot diameter: $r_{spot} = \frac{2*M^2*\lambda*f_{Obj}}{\pi*D_{laser}}$

Depth of field: $DoF = 2 * \frac{\pi*{r_{spot}}^2}{M^2*\lambda}$

6. PCA-loadings of the Raman spectra used for class separation

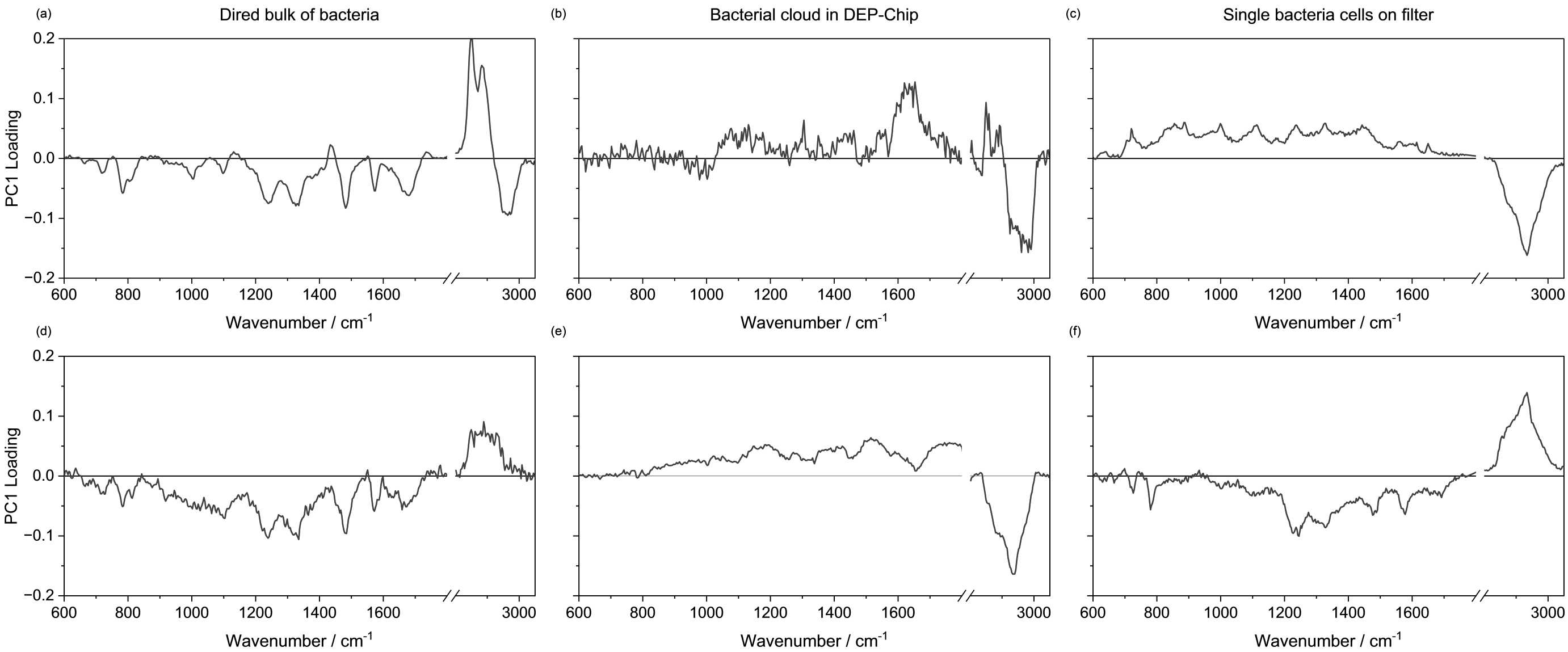

Figure SI 6.1 PC1 loadings of the Raman spectra used for class separation**.** The first principal component loadings (PC1) obtained from the PCA performed on the difference spectra are shown for the different Raman measurement strategies and antibiotic treatments. Panels (a–c) correspond to levofloxacin treatment and panels (d–f) to amoxicillin treatment. Within each row the measurement strategies are ordered as follows: (a,d) dried bulk, (b,e) DEP-chip, and (c,f) single-cell.

7. Resistance testing of Levofloxacin on the DEP-Chip with 785 nm

Due to the poor spectral quality observed in several measurements using 532 nm, the same biological samples were additionally measured using 785 nm excitation on the inverse WITec alpha300 Ri system on two measurement days. Using the spectra obtained with 785 nm excitation, the PCA-LDA model showed a clearer separation between the phenotypic classes “growth” and “no growth” (Figure SI 7.1). At the spectral level, the model achieved a balanced accuracy of 84.7 %. When the replicated spectra were aggregated at the batch level using a majority decision approach, the classification performance improved further, resulting in a perfect separation between the two classes, corresponding to a balanced accuracy of 100 % (Table SI 7.1). These results suggest that, under the experimental conditions used, excitation at 785 nm allowed for a clearer distinction between the phenotypic responses induced by antibiotics. However, as these additional measurements were only available for two batches, the results should be interpreted with caution and should not be taken as evidence of a generally superior performance of excitation at 785 nm.

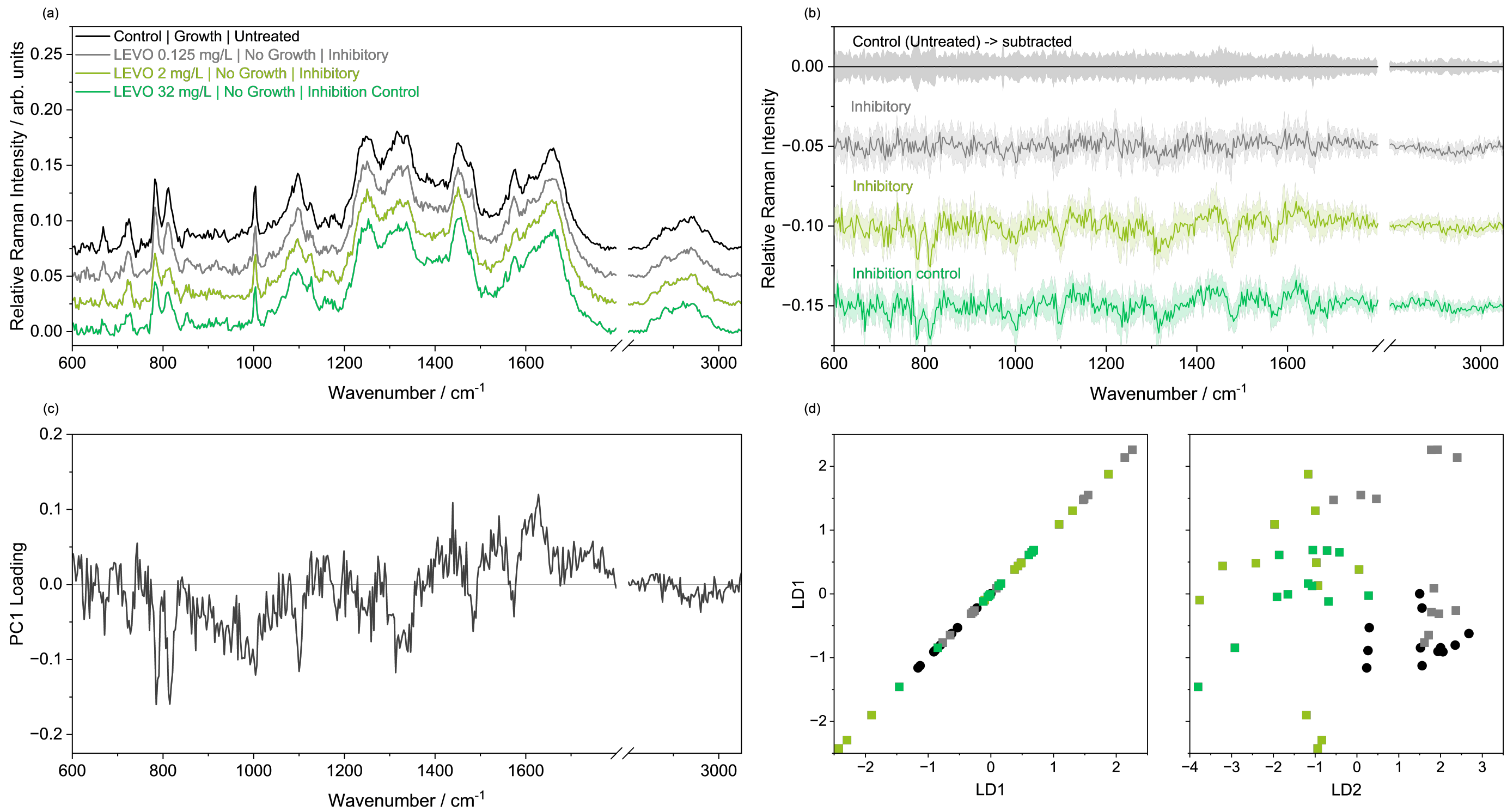

Figure SI 7.1 Raman-based analysis of the phenotypic response of *E. coli* to levofloxacin, measured at an excitation wavelength of 785 nm on the inverse WITec alpha300 Ri system. (a) Preprocessed mean Raman spectra of untreated bacteria and cells exposed to inhibitory concentrations of levofloxacin, shown in the respective colors. (b) Corresponding difference spectra, obtained by subtracting the mean Raman spectrum of the untreated control from the spectra recorded under antibiotic exposure. (c) Corresponding PCA-loading of the first principal component. (d) Linear discriminant analysis (LDA) scores, showing the separation of spectra assigned to the phenotypic classes “growth” and “no growth”.

Table SI 7.1 PCA-LDA model for predicting the two classes "growth" and "no growth" at spectral and batch level when treated with levofloxacin.

**Spectral level**		**Predicted**		**Balanced accuracy**
		No Growth	Growth	
True	No Growth	27	9	**83.3 %**
	Growth	1	11	
**Batch level**		**Predicted**		**Balanced accuracy**
		No Growth	Growth	
True	No Growth	2	0	**100 %**
	Growth	0	2	

References

1. EUCAST. The European Committee on Antimicrobial Susceptibility Testing. Breakpoint tables for interpretation of MICs and zone diameters. Version 16.0. 2026.

2. ThorLabs. Imaging Microscope Objectives. 2026. https://www.thorlabs.com/imaging-microscope-objectives?tabName=Objective%20Tutorial. Accessed 22 Mar 2026.
